# Effects of Resveratrol on Inflammatory Biomarkers in Glaucomatous Human Trabecular Meshwork Cells

**DOI:** 10.3390/nu11050984

**Published:** 2019-04-30

**Authors:** Selom Avotri, Danita Eatman, Karen Russell-Randall

**Affiliations:** 1 Department of Pharmacology & Toxicology, Morehouse School of Medicine, 720 Westview Drive, SW, Atlanta, GA 30310, USA; SAvotri@alumni.msm.edu; 2 Department of Medical Education, Morehouse School of Medicine, 720 Westview Drive, SW, Atlanta, GA 30310, USA; DEatman@msm.edu; 3 Department of Microbiology, Biochemistry & Immunology, Morehouse School of Medicine, 720 Westview Drive, SW, Atlanta, GA 30310, USA

**Keywords:** resveratrol, GTM-3 cells, nitric oxide, eye

## Abstract

Purpose: Resveratrol (RSV), an antioxidant polyphenol, has demonstrated beneficial effects in various ocular diseases including glaucoma. Our study was designed to evaluate the effects of RSV on nitric oxide synthase (NOS) enzymes, nitric oxide (NO) and interleukin-1 alpha (IL-1 α), in human glaucomatous trabecular meshwork (TM) cells. Methods: Western blot was utilized to determine endothelial and inducible NOS (eNOS, iNOS) expression. The concentration-related effects of RSV on IL-1 α and NO levels were assessed using the respective ELISA kits. Results: Densitometry data showed concentration-related increases in eNOS, and reduction in iNOS expression at high RSV concentrations. RSV treatment (0.1, 1, 10 and 100 µM) resulted in increased NO levels (6 ± 0.7, 7 ± 0.8, 7.3 ± 0.7 and 9.5 ± 1 nM/mg protein, respectively). The average value obtained for control was 4.8 ± 0.6 nM/mg protein. Significant increases in IL-1α levels were observed with lower concentrations of RSV. However, at higher RSV concentrations (10–100 μM), IL-1 levels decreased. Conclusions: Resveratrol increased NO in glaucomatous TM cells, possibly by increasing eNOS expression. Thus, RSV-induced NO production supports the beneficial effects of this antioxidant in glaucoma. Furthermore, our results showing a reduction in iNOS, a contributor to oxidative stress expression, further support RSV’s antioxidant capabilities in vision.

## 1. Introduction

Glaucoma is a neurodegenerative disease often associated with age progression. Progressive visual field loss and possible irreversible blindness accompany this ocular disease [1]. There are multiple factors that may contribute to the initiation and progression of glaucoma, such as increased circulating glutamate levels, alternations in nitric oxide (NO) metabolism, vascular alterations, oxidative stress, and inflammation [2]. Our laboratory is interested in the oxidative stress-induced degeneration of the ocular epithelium in the trabecular meshwork (TM) endothelial cells, which is a characteristic of primary open-angle glaucoma (POAG). The TM plays a vital role in the regulation of aqueous humor dynamics and the maintenance of intraocular pressure (IOP). In POAG patients, the number of TM endothelial cells is significantly decreased when compared to age-matched controls [2]. Oxidative stress and inflammation are believed to be primary factors in the loss of these TM cells [2,3]. The oxidative stress can also cause DNA damage, which is a significant factor inducing elevated IOP in POAG [1]. Together, high intraocular pressure and aging serve as the main risk factors for glaucoma. 

With the association between oxidative stress and age-related disease, phytochemicals, which have anti-inflammatory and antioxidant properties, have been studied as potential preventative treatments for ocular diseases. Resveratrol, the most studied phytochemical, has been identified as a possible preventative agent for the aging eye. Resveratrol is an organic component that is naturally found in plants, such as grapes, peanuts, and berries [4] and has a range of biological effects, such as cardiovascular protection and neuroprotection properties [5,6]. The protective effects of resveratrol within the eye are extensive. Several studies have reported findings that support the effect of resveratrol on several pathways, including oxidative stress and inflammation, which are involved in a number of eye diseases [4]. The molecular mechanisms underlying these protective actions of resveratrol are not well defined.

Deciphering the cellular mechanisms by which resveratrol protects against aging diseases is of interest to our lab. Several investigators have reported that resveratrol reduction in pro-inflammatory TNF-alpha and interleukin 8 cytokines may serve as a possible mechanism of action for reducing oxidative stress [7]. Oxidative stress and inflammatory markers, such as nitric oxide, ELAM-1, and IL-1 α, are some of the factors that have been evaluated using animal and human trabecular meshwork (TM) cells. In glaucomatous TM cells, constitutive expression of IL-1 α has also been observed and leads to the upregulation of ELAM-1 and other inflammatory markers [8]. Because of the many physiologic changes that have been found in glaucoma, studying variations in NO expression on TM inflammation after resveratrol treatment may provide clues to improving TM aqueous humor outflow pathway resistance. Therefore, we hypothesized that resveratrol would increase NO production and reduce specific inflammatory markers in human glaucomatous cells. Thus, we analyzed the effects of resveratrol treatment on nitric oxide levels and the expression of selected markers for inflammation in GTM-3 cells.

## 2. Materials and Methods

### 2.1. Cell Culture

Human glaucomatous trabecular meshwork cells (GTM-3), a kind gift from Alcon Research Laboratories, Fort Worth, TX, USA (now Novartis), were stored in liquid nitrogen or at −80 °C and maintained in high glucose Dulbecco’s modified Eagle medium with L-glutamine, 10% fetal bovine serum (FBS), 1% penicillin/streptomycin, and 0.1% amphotericin B, an antifungal agent. Cells were thawed to 32 °C and mixed with fresh media before being incubated. Cells were incubated at 37 °C, 5% CO_2_, and 95% relative humidity for 48 h. Cells were visualized under a microscope using the Zeiss Axiovert 200M (Carl Zeiss, Göttingen, Germany).

### 2.2. Resveratrol Treatment

Once confluent, GTM-3 cells were rinsed with warm phosphate buffered saline (PBS, ×2) before trypsinizing. Trypsin was neutralized with fresh media at double the volume and centrifuged at low speed for 5 min. Media was then replaced with warm PBS and centrifuged for 5 min and repeated once. Fresh media (2 mL) was then added to each dish. The cells were plated in 60 cm^2^ dishes, with each treatment being done in triplicate. Upon growth to approximately 90% confluent monolayer, the growth media was replaced with phenol-free DMEM media containing the respective concentrations of resveratrol. Resveratrol was dissolved in DMSO at concentrations of 0.1, 1, 10, and 100 μM, respectively. Control cells contained the same concentration of DMSO as the cells treated with the highest concentration of resveratrol in culture media. This concentration did not exceed 0.01% DMSO. 

### 2.3. Western Blot Analysis

Western blot was performed to determine the protein expression of eNOS and ELAM-1. Cell cultures were treated with resveratrol for 24 h, washed with PBS, and lysed using ice-cold radioimmunoprecipitation assay (RIPA) buffer (Teknova, Hollister, CA, USA) with Halt protease inhibitor (ThermoFisher Scientific, Grand Island, NY, USA). Cells were scraped and the cell lysates were then suspended for 15 min by triturating on ice before being centrifuged at 14,000 g for 15 min, 4 °C. The supernatant was aliquoted and stored at −80 °C until use. 

Protein concentration was determined using a BSA protein assay (Bio-Rad, Hercules, CA, USA), following the microassay procedure for microtiter plate protocol. Five dilutions of protein standards were prepared in duplicates for a linear range of the assay between 8 μg/mL to 80 μg/ mL. Each standard and sample solution (160 μL) was then added to separate wells in duplicates followed by the addition of dye reagent (40 μL) for absorbance analysis at 595 nm using Softmax Pro software (Molecular Devices, San Jose, CA, USA). 

Invitrogen WesternBreeze chemiluminescent immunodetection kits (ThermoFisher Scientific, Grand Island, NY, USA) were used to complete electrophoresis, transfer, and blot. The various samples were prepared by following protocol for reduced samples provided with the NuPAGE Novex Bis-Tris 4–12% gradient gels. Standard western blot procedures were followed using primary antibodies for ELAM-1 (1:50), eNOS (1:200) and iNOS (1:200). Negative controls were primary antibodies that were prepared in a 1:1 ratio with blocking peptide. Proteins were visualized by briefly incubating membranes in chemiluminescence substrate for 5 min before exposing to X-ray film at various exposure times. The protein densities were compared to GAPDH protein loading control to obtain corrected values for quantification of Western blot data. 

### 2.4. IL-1 α Evaluation

The levels of the pro-inflammatory biomarker IL-1 α was determined by ELISA kit (Invitrogen, ThermoFisher Scientific, Grand Island, NY, USA). The protocol was followed according to the instructions provided in the kit. Absorbance was read at 450 nm against chromagen blanks using Softmax Pro (Molecular Devices, San Jose, CA, USA).

### 2.5. Nitric Oxide Evaluation

NO levels were determined using the Active Motif Nitric Oxide Quantitation kit (Active Motif, Carlsbad, CA, USA) by following the included protocol for cell lysate samples. Absorbance was measured at 540 nm with a reference wavelength of 620 nm.

Control samples were collected at 0 h. prior to resveratrol treatment. After resveratrol treatment (100 μM), samples were collected at 2, 4, 8, 12, and 24 h before completing the NO assay. GTM-3 cells were plated in 60 mm^2^ dishes and grown to confluence. L-arginine dissolved in PBS was added to DMEM media at 10^−2^ M to stimulate NO synthesis for quantification of NO levels before resveratrol treatment for both NO studies. L-arginine was used because NO synthesis occurs through metabolism of L-arginine. 

Untreated, (vehicle only) and positive controls were utilized in these experiments. The positive control was established to validate the proper function of the system. Estradiol served as the positive control because it is known to significantly increase levels of NO in all systems [9]. The vehicle control groups in this experiment were treated with 0.01% DMSO and estradiol was dissolved in 0.01% DMSO for positive control treatments and added to DMEM media at a final concentration of 10^−4^ M. 

### 2.6. Statistical Analysis

Results are presented using the mean ± SEM of at least three experiments. Statistical analyses were performed using SigmaPlot 12.0 Software (Systat Software, Inc., San Jose, CA, USA) and Microsoft Excel (Microsoft Corporation, Redmond, WA, USA). Statistical significance was obtained using analysis of variance test (ANOVA) post hoc tests (Dunnett’s test, Dunn’s test, Student–Newman–Keuls method, and Holm–Sidak method). *p* values of < 0.05 were considered statistically significant.

## 3. Results

### 3.1. Resveratrol does Not Prevent Cell Proliferation 

**Figure 1:**
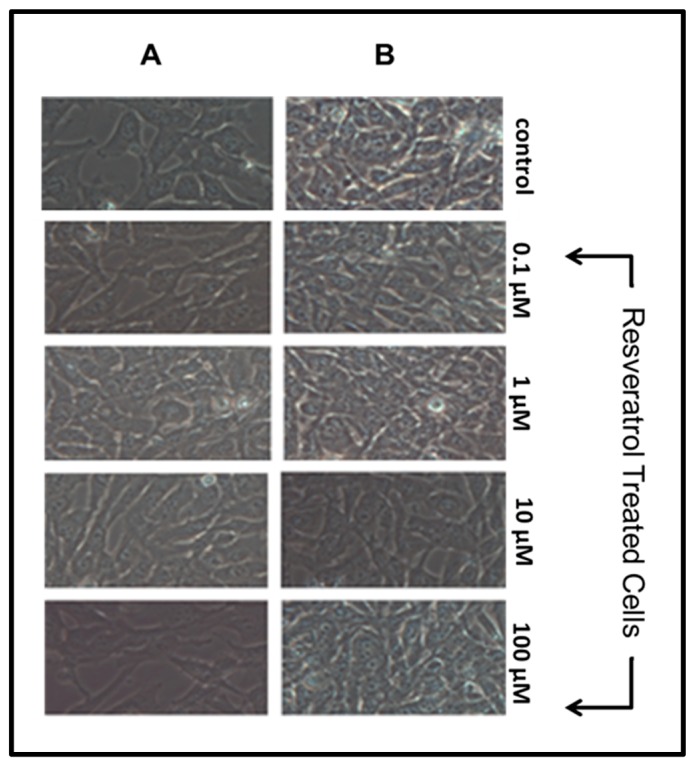
GTM-3 cells prior to treatment conditions (**A**) and 24 h after treatment (**B**) (*n* = 3).

We observed the different treatment conditions through visual inspection via the Zeiss Axiovert 200 M. Resveratrol did not have an effect on cell proliferation or lead to apoptosis after 24 h. Column A (Figure 1) illustrates normal cell proliferation prior to resveratrol treatment. As cells proliferate, they take a flat shape and adhere to culture plate surfaces. It is essential for nearby cells to make contact for proper proliferation. 

Column B (Figure 1) illustrates normal cell proliferation following 24 h exposure to resveratrol at various concentrations. Instances of cell damage or apoptosis are characterized by a change in morphology of GTM-3 cells (a rounding shape) as well as cell detachment from culture plate surfaces. Our results are consistent with previous studies that found no direct damaging effects of resveratrol on TM cells at concentrations under 200 μM [8]. Some studies have shown damaging effects of resveratrol at higher concentrations [8], yet preliminary studies conducted in our lab did not exhibit damaging effects of resveratrol treatment at 200 μM.

### 3.2. The Effects of Resveratrol on NO Levels in GTM-3 Cells 

**Figure 2:**
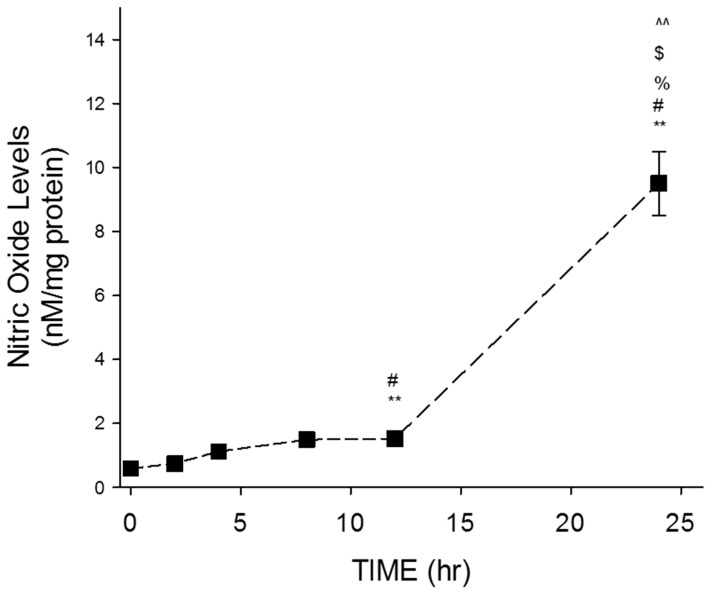
Time-dependent effects of resveratrol on NO levels in GTM-3 cells. Figure shows mean ± SEM (*n* = 3). ** = *p* < 0.05 compared to 0 h, # = *p* < 0.05 compared to 2 h, % = *p* < 0.05 compared to 4 h, $ = *p* < 0.05 compared to 8 h, ^^ = *p* < 0.05 compared to 12 h.

Time-dependent evaluation of NO levels following resveratrol treatment was performed to determine the most effective treatment time (Figure 2). The highest concentration of resveratrol (100 μM) was utilized in these experiments. 

Results indicate a time-dependent increase in NO levels over the course of 24 h (Figure 2). The average NO values obtained for 0, 2, 4, and 8 h were 0.59 ± 0.19, 0.74 ± 0.08, 1.12 ± 0.11, and 1.49 ± 0.13, respectively. Significant increases in NO levels were exhibited at 12 h (1.5 ± 0.1), while the average NO value obtained for 24 h was 9.5 ± 1. Obtained average NO values were represented in units of nM/mg protein. 

### 3.3. Concentration-Dependent Effects of Resveratrol on NO Levels in GTM-3 Cells 

Evaluation of NO levels in GTM-3 cells following resveratrol treatment at various concentrations for 24 h was performed to evaluate if changes in NO levels occurred in a concentration-dependent manner. 

**Figure 3:**
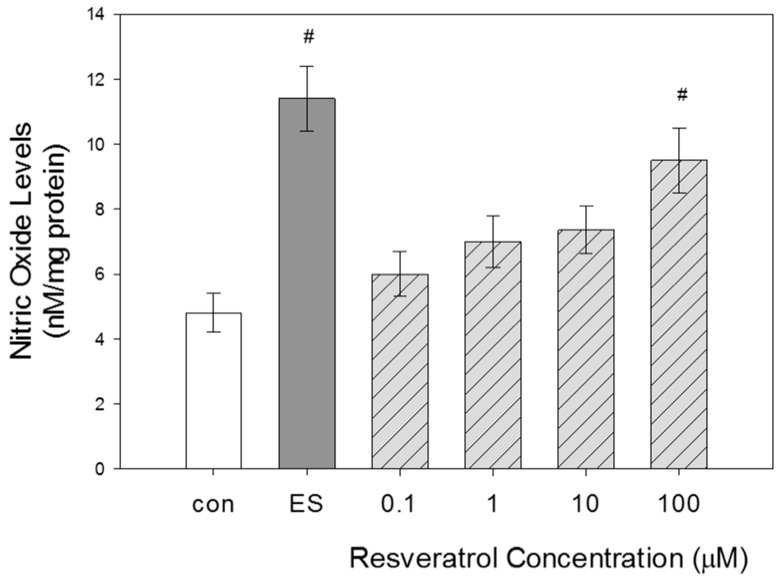
Concentration-dependent effects of resveratrol on NO levels in GTM-3 cells. Resveratrol treatment is at different concentrations for 24 h (*n* = 3). ES = estradiol. Figure shows mean ± SEM. # = *p* < 0.05 compared to control. con—control.

As expected, estradiol treatment yielded significant increases in NO levels when compared with control. This result validated proper functioning of the system. Our results indicate that NO levels increased with increasing concentration of resveratrol (Figure 3). The most significant increase in NO levels occurred at 100 μM. The average value obtained for the control group was 4.8 ± 0.6. The average value obtained for estradiol-treated cells was 11.4 ± 1. In increasing order of resveratrol concentration at 0.1, 1, 10 and 100 µM, the average values obtained were 6 ± 0.7, 7 ± 0.8, 7.3 ± 0.7 and 9.5 ± 1, respectively. It was expected that the highest concentration of resveratrol, 100 μM, would yield the highest NO levels because this concentration also yielded the highest increase in eNOS expression.

### 3.4. The Effects of Resveratrol on eNOS Expression in GTM-3 Cells

To evaluate the effects of resveratrol on eNOS protein expression, western blot analyses were performed following 24 h of resveratrol treatment. Our data indicate a strong band at 137 kDA in molecular weight. The control group of untreated cells and the lowest concentration of 0.1 μM yielded the lowest expression of eNOS. 

**Figure 4:**
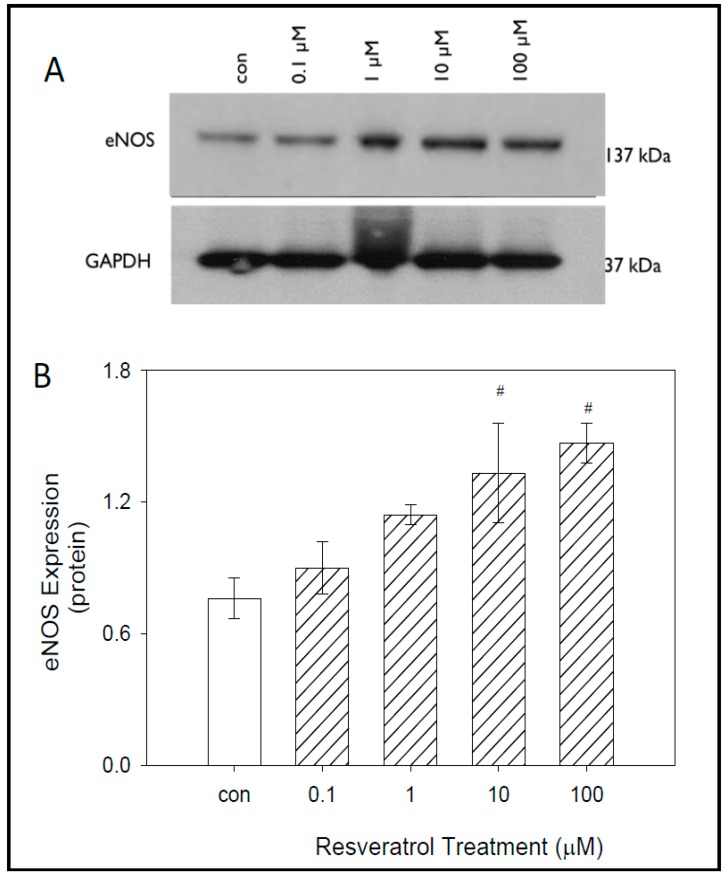
Effects of resveratrol on eNOS expression in GTM-3 cells. Figure shows mean ± SEM (*n* = 3). # = *p* < 0.05 compared to control. con—control.

As expected, results indicate increasing eNOS expression with increasing resveratrol concentration. Higher concentrations of resveratrol treatment yielded significantly higher expression of eNOS in GTM3 cells. GAPDH loading control was used to confirm accurate protein loading (Figure 4). 

Densitometry data confirmed that increased eNOS expression occurred in a concentration-dependent manner (Figure 4). At control, 0.1 μM, and 1 μM, the average protein values were 0.76 ± 0.09, 0.90 ± 0.12, and 1.14 ± 0.04 respectively. Significant increases in eNOS expression were observed at resveratrol concentrations of 10 μM and 100 μM. At 10 μM an average protein value of 1.33 ± 0.23 was obtained, while at 100 μM an average protein value of 1.47 ± 0.09 was obtained as compared with control. Both 10 μM and 100 μM have average protein values almost two-fold when compared with control. All protein values obtained are represented in arbitrary units.

### 3.5. The Effects of Resveratrol on iNOS Protein Expression in GTM-3 Cells

**Figure 5:**
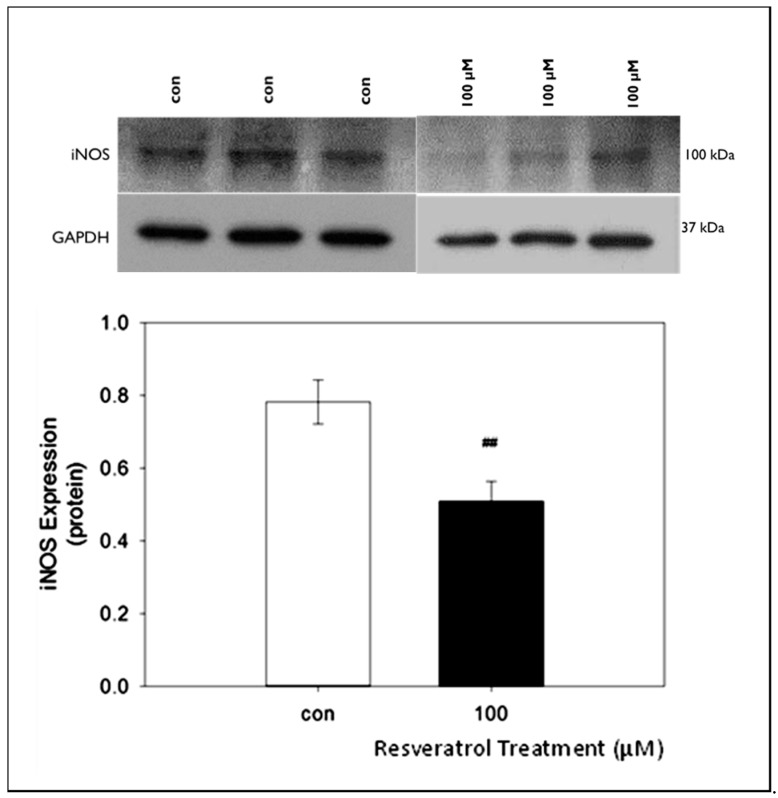
The effects of 100 µM resveratrol concentration on iNOS expression in GTM-3 cells. Figure shows mean ± SEM (*n* = 3). ## = *p* < 0.05 compared to control. con—control.

We also examined the changes in iNOS protein expression following treatment with resveratrol at the highest concentration used in the other studies. Our data consistently indicate strong bands at approximately 100 kDA (Figure 5). 

Quantification of western blot data yielded average protein values of 0.78 ± 0.06 and 0.51 ± 0.05 for control and 100 μM resveratrol, respectively. Our results indicate a significant decrease in iNOS concentration at resveratrol concentration of 100 μM (Figure 5).

### 3.6. The Effects of Resveratrol on IL-1 α Expression in GTM-3 Cells 

**Figure 6:**
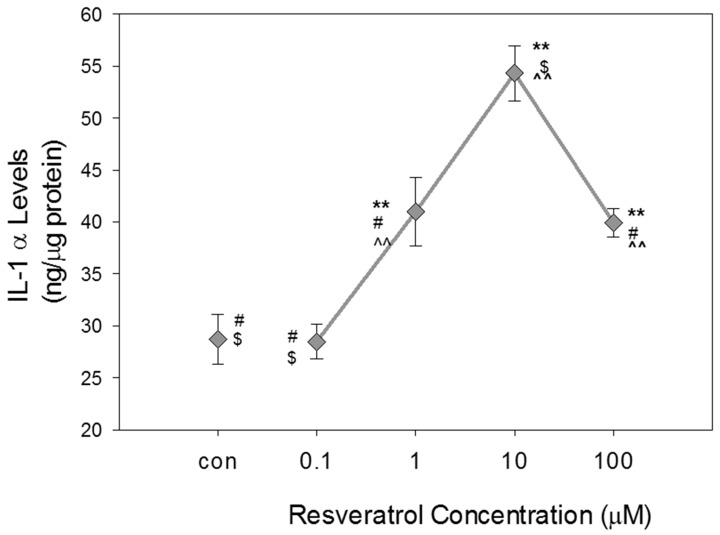
Effects of resveratrol on IL-1 α levels in GTM-3 cells following 24-h exposure. Figure shows mean ± SEM (*n* = 3). ** = *p* < 0.05 compared to control, ^^ = *p* < 0.05 compared to 0.1 µM, $ = *p* < 0.05 compared to 1 µM, # = *p* < 0.05 compared to 10 µM. con—control.

IL-1 α was evaluated using ELISA to determine if resveratrol had any effects on this inflammatory biomarker. Significant increases in IL-1 α levels were observed with increasing concentrations of resveratrol up to a concentration of 10 μM (Figure 6). The average value obtained for the control was 28.7 ± 2.4. The average value obtained for 0.1 μM was 28.5 ± 1.6. The values obtained for concentrations of 1, 10, and 100 μM were significantly different compared to the control. The respective concentrations had average values of 41 ± 3, 54 ± 3, and 40 ± 1.

Treatment of cells with resveratrol showed increases in IL-1 α levels that reached a maximum level at 10 μM before significantly decreasing at 100 μM resveratrol treatment. Average values were represented in units of ng/µg of protein. 

## 4. Discussion

Elevated intraocular pressure (IOP) is often described as the most prominent risk factor in primary open-angle glaucoma (POAG), an age-related optic neuropathy that affects millions of people, and is the main cause of irreversible blindness worldwide. The damage to the retina and optic nerve head seen in POAG is due, in part, to elevated intraocular pressure (IOP, ocular hypertension). This increased IOP is believed to be due to damage to trabecular meshwork (TM) tissue that is responsible for draining aqueous humor from the anterior chamber of the eye. The TM plays a vital role in anterior chamber fluid regulation and in keeping IOP at a steady state. 

Many factors may contribute to the ocular hypertension observed in POAG, but oxidative stress and inflammation have been extensively studied and suggested as factors causing the high IOP. Oxidative stress has been shown to be a driving force for the loss of TM endothelial cells in POAG [2,3]. Oxidative stress and inflammatory markers, such as nitric oxide, ELAM-1, and IL-1 α, are some of the factors that have been evaluated using animal and human TM cells. Previous studies on porcine TM cells that experienced induced oxidative stress showed beneficial effects of resveratrol [8]. Thus, this study was designed to examine inflammatory biomarker expression and levels in glaucomatous TM cells following resveratrol treatment. Our data demonstrate that resveratrol treatment caused a concentration-dependent increase in NO levels, eNOS protein expression and IL-1 α levels in human glaucomatous TM cells. Resveratrol also caused a significant reduction in iNOS protein expression at the highest concentration of RSV (100µM) used in this study. 

To our knowledge, the physiological levels of RSV in the aqueous humor are unknown. In a study by Wang et al. [10] in which patients were given a nutritional supplement containing 100 mg of trans-RSV, the actual RSV levels were shown to be below the detectable limits of the assay, but the metabolites were at levels between 86.11 and 1503.76 nmol/L in the aqueous humor. Based on this information, it is likely that the concentrations of RSV (0.1–100 μM) used in our study were within, and possibly higher (100 μM) than, the expected physiological levels. 

There is a paucity of data on the specific antioxidant effects of RSV on age-related ocular diseases in humans, but there is evidence that an RSV nutritional supplement produced beneficial effects on visual function in age-related macular degeneration (AMD) patients [11]. More research is needed to confirm the beneficial effects of RSV on age-related ocular diseases, particularly glaucoma, in humans. Studies in a rat model of glaucoma [12], as well as in vitro studies, suggest that RSV has ocular hypotensive effects and protects retinal and trabecular meshwork cells [8,13,14]. These data imply that RSV may have a role in the management and/or prevention of ocular diseases caused by damage to these specific cells. Our data support this hypothesis.

Resveratrol’s effects have been studied in retinal pigment epithelium (RPE) and in porcine and human TM cells, and has been shown to exhibit beneficial effects on oxidative stress, inflammation, and apoptosis. Furthermore, studies have not found cytotoxic effects or cell damage and reduced viability at concentrations below 200 µM [8]. At high concentrations of RSV (200 μM and 400 μM), TM cells experienced extensive cell death in less than 48 h [8]. However, in our studies, glaucomatous TM cells treated with 200 μM of resveratrol for 24 h did not exhibit cytotoxic effects. The differences in results following higher concentration of resveratrol exposure may be due to the fact that the cells in the earlier study were primary porcine TM cells, while the cells in our study were transformed glaucomatous human TM cells that were isolated 24 h post mortem from a 72-year-old male POAG patient [15]. To our knowledge, these cells are currently the only glaucomatous TM cell line available. The other TM cell lines, TM-1 and NTM5, were isolated from non-glaucomatous donors. The phenotype of the GTM3 cells differs from that of primary human TM cells in that they are smaller, do not have an extensive cytoskeleton and do not make as much extracellular matrix as primary cultures [16], but they are useful models for extensive biochemical and pharmacological studies. 

We demonstrate that the effect of RSV on glaucomatous TM cells was time-dependent. The levels of NO released by these cells increased over time with the most significant release observed at 24 h. This finding served as the basis for treatment time on all subsequent studies. NO is produced through the metabolism of L-arginine. This is catalyzed by the enzyme eNOS, iNOS, or nNOS. The specific NOS isoform that is utilized for NO production is dependent on the location where NO production is necessary. The expression of all three isoforms is present in various structures of the eye that regulate the flow of aqueous humor. In our studies, we first evaluated the expression of eNOS because the TM is composed of endothelial-like cells disbursed between collagen lamellae and elastic fibers. Our results demonstrate that resveratrol produced a concentration-dependent increase in eNOS expression in glaucomatous TM cells. Our results in the endothelial-like TM cells are consistent with data observed in other studies where resveratrol was shown to increase eNOS activity in human endothelial cells [17]. The most significant increases in eNOS were at concentrations of 10 μM and 100 μM. Based on this data, we predicted that resveratrol would increase NO levels in GTM-3 cells and that the maximum effect would be at 100 μM. Upon evaluation of NO levels, we observed a concentration-dependent increase of NO levels with a maximum increase at 100 μM, consistent with the concentration-dependent increase of expression in eNOS. Altogether, these data show that increased activity of eNOS following resveratrol possibly contributed to increased levels of NO in GTM-3 cells. 

Studies from Ren-yi et al. [18] suggest that activation of iNOS levels in TM cells may result in negative effects due to the accumulation of peroxynitrite and nitrite as a result of free radical formation in the tissues that could lead to further DNA damage, cell apoptosis, neurotoxicity, and inflammation of the tissue. We evaluated iNOS protein expression in GTM-3 cells following resveratrol treatment to determine if iNOS expression is affected by resveratrol. We chose the highest concentration of resveratrol because our previous results from studies on eNOS expression and NO levels consistently showed significant changes at 100 μM. Our data show a significant decrease in iNOS protein expression following resveratrol treatment when compared to control. This confirms that the increased NO levels observed following resveratrol treatment in GTM-3 cells are not due to an increase in iNOS protein expression. Since studies suggest iNOS activation as a potential contributor to oxidative stress, our results showing a reduction in iNOS expression support previous studies listing resveratrol as having anti-oxidant capabilities.

RSV treatment has been shown to reduce pro-inflammatory cytokines such as tumor necrosis factor–alpha (TNF-α), and interleukin–1 beta (IL-1β). In a recent study, Ahmad et al. [19] documented the neuroprotective effect of RSV and reported that RSV treatment decreased TNF-α expression in mouse brain tissue. In addition, IL-6 mRNA and protein levels were attenuated in the presence of RSV in the mouse brain. Another report revealed that RSV reduced TNF-α and IL-1β expression in glial and motor neuron cells [20]. Based on these findings and the regulation of iNOS by TNF-α, it is possible that the reduction in iNOS expression observed in our study may be due to the inhibition of TNF-α by RSV. Additional studies are needed to confirm this association.

NO is a key component produced by the body that increases blood flow which helps carry oxygen and needed nutrients to muscles. Some studies have suggested that reduced blood flow contributes to the pathogenesis of glaucoma [1]. This finding supports the importance of NO in GTM cells. Thus, resveratrol may serve as a beneficial agent In POAG because increases in NO levels following treatment could be extrapolated to contribute to lowering IOP through improved contractility of TM cells allowing for improved aqueous humor outflow. NO may also locally assist in vascular regulation by increasing vasodilation and increasing ocular blood flow. If NO increases both aqueous humor outflow and ocular blood flow, glaucomatous symptoms in the TM, as well as other structures of the aqueous humor outflow pathway may decrease. 

Various studies have found that the autocrine feedback loop initiated through IL-1 activation contributes to a stress-response in damaged or altered cells. Autocrine feedback loops can be activated in tissue repair, disease, and cellular aging, and serve to amplify expression of specific genes [21]. In glaucomatous TM cells, activation of the autocrine feedback loop initiates the transcription factor NF-κB to activate inflammatory cytokines such as IL-1 α, IL-1 β, IL-6, IL-8, and ELAM-1. In our studies, resveratrol increased IL-1 α levels in glaucomatous TM cells. Interestingly, the concentration-dependent increase in IL-1 tended to decline at high resveratrol concentration of 100 μM, suggesting that IL-1 α may have a maximum threshold response at 10 μM. In a previous study, Luna et al. [8] observed decreases in IL-1 α expression and ELAM-1 expression in primary porcine TM cells subjected to induced oxidative stress. It must be noted that the disease model utilized in this study was TM cells that were transformed from a patient suffering from POAG, so this may be a factor on why a decrease in IL-1 α was not observed. Additionally, the difference in cell model may account for differences in our findings to those reported by Luna et al. [8]. We expected that resveratrol would decrease IL-1 α levels as a result of increased NO levels in GTM-3 cells to provide show that resveratrol reduces inflammation. However, in glaucomatous TM cells, it has been suggested that IL-1 is produced endogenously to inhibit the apoptotic response to oxidative stress. Thus, we suspect that the increase in IL-1 α observed in this study in response to resveratrol at low concentration may inhibit cell death. Further studies are needed to determine if this increase in IL-1 α levels contributes to an initial protective stress response or if the increases contribute to sustained activation of the stress-response that contributes to negative effects on TM cells. 

Li et al. [22] suggested that sub-lethal oxidative damage induces inflammatory markers but also leads to prolonged increases in the endogenous generation of iROS in several cell types. This iROS generation has the potential to result in sustained activation of NF-κB, which can lead to constitutive activation of the stress response that results in pathologic effects and contribute to the progression of glaucoma. In addition, upon studying contributors to iROS generation, they determined that eNOS did not significantly contribute to the increase in iROS production. This finding supports our findings as it relates to resveratrol increasing eNOS expression in GTM-3 cells as a mechanism for increasing NO levels. This increase in NO levels may also contribute to beneficial effects on relaxing TM cells without contributing to the formation of iROS species which could present negative effects. 

Although ELAM-1 expression was shown to decrease in a previous study [8] using primary porcine TM cells subjected to induced oxidative stress, our data show no changes with resveratrol treatment (data not shown). We predicted that resveratrol would decrease ELAM-1 expression in GTM-3. Our studies indicate that resveratrol had no significant effect on altering ELAM-1 expression. This indicates that maybe resveratrol-induced increases in NO levels in GTM-3 cell do not directly correlate to changes in ELAM-1 expression. The results could also indicate that the concentration of resveratrol used in these experiments was not high enough to have an effect on the already high levels of ELAM-1 in these cells. Taken together, it is possible that resveratrol treatment may provide beneficial effects on eNOS expression and NO levels, but may not change the expression of ELAM-1 in cells that are already glaucomatous. Furthermore, this may suggest that resveratrol treatment is more beneficial as a preventative measure in patients that do not already exhibit symptoms of POAG or in patients that detect early symptoms of POAG. 

## 5. Conclusions

**Figure 7:**
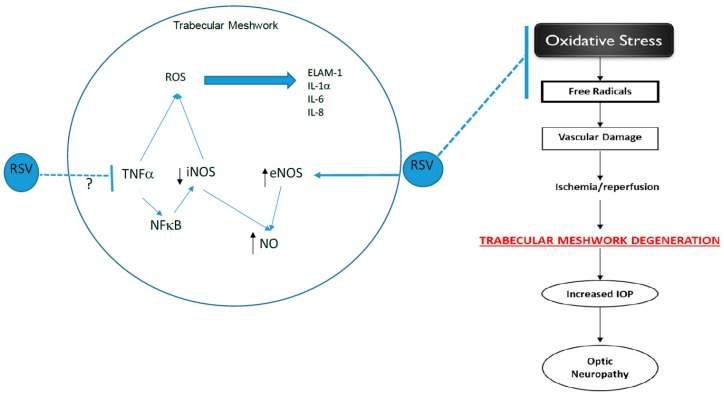
Schematic overview of the mechanisms underlying the beneficial effects of resveratrol on the trabecular meshwork (TM). Our data demonstrated that RSV inhibited iNOS expression but increased eNOS and NO levels in TM cells. We suspect that RSV may inhibit iNOS by blocking TNF-α; thus, preventing vascular damage, which ultimately leads to TM degeneration. RSV—resveratrol; iNOS—inducible nitric oxide synthase; eNOS—endothelial nitric oxide synthase; NO—nitric oxide; ROS—reactive oxygen species, ↑—increased activity, ↓—decreased activity.

In conclusion, the results obtained from this study indicate that resveratrol increased NO levels, possibly by increasing eNOS activity in glaucomatous TM cells. The anti-inflammatory effects of resveratrol in these cells were demonstrated by its reduction of iNOS protein expression at a concentration that also caused a reduction in IL-1 α levels. Resveratrol also caused changes in IL-1 α levels that did not correlate to ELAM-1 expression. Although both IL-1 influences ELAM-1 through an autocrine feedback loop, the fact that ELAM-1 expression did not follow the same trend as IL-1 α indicates that there may be other mechanisms or signaling pathways that also affect ELAM-1 expression in glaucomatous TM cells. Furthermore, the progression of the disease may also be a determining factor of ELAM-1 expression. The anti-oxidant, anti-inflammatory, and anti-apoptotic capabilities of resveratrol have shown the beneficial effects of resveratrol in TM cells. Our study supports the use of resveratrol as an agent to increase NO production in vitro in TM cells. Decreased NO production in TM cells has been found to be a risk factor for POAG, and increasing NO production as a result of resveratrol supports the beneficial effects of resveratrol in these cells. Figure 7 shows an overview of the possible mechanisms underlying the beneficial effects of resveratrol on TM, thus ultimately protecting the aging eye.
